# Maternal smoking during pregnancy and type 1 diabetes in the offspring: a nationwide register-based study with family-based designs

**DOI:** 10.1186/s12916-022-02447-5

**Published:** 2022-08-12

**Authors:** Yuxia Wei, Tomas Andersson, Jessica Edstorp, Josefin E. Löfvenborg, Mats Talbäck, Maria Feychting, Sofia Carlsson

**Affiliations:** 1 Institute of Environmental Medicine, Karolinska Institutet, Nobels väg 13, 17177 Stockholm, Sweden; 2 Centre for Occupational and Environmental Medicine, Stockholm County Council, Stockholm, Sweden

**Keywords:** Type 1 diabetes, Siblings, Perinatal epidemiology, Confounding factors

## Abstract

**Background:**

Maternal smoking during pregnancy was reported to be associated with a reduced risk of type 1 diabetes in the offspring. We investigated whether this association is consistent with a causal interpretation by accounting for familial (shared genetic and environmental) factors using family-based, quasi-experimental designs.

**Methods:**

We included 2,995,321 children born in Sweden between 1983 and 2014 and followed them for a diagnosis of type 1 diabetes until 2020 through the National Patient, Diabetes and Prescribed Drug Registers. Apart from conducting a traditional cohort study, we performed a nested case–control study (quasi-experiment) comparing children with type 1 diabetes to their age-matched siblings (or cousins). Information on maternal smoking during pregnancy was retrieved from the Swedish Medical Birth Register. Multivariable adjusted Cox proportional hazards regression and conditional logistic regression were used.

**Results:**

A total of 18,617 children developed type 1 diabetes, with a median age at diagnosis of 9.4 years. The sibling and cousin comparison design included 14,284 and 7988 of these children, respectively. Maternal smoking during pregnancy was associated with a 22% lower risk of offspring type 1 diabetes in the full cohort (hazard ratio 0.78, 95% confidence interval [CI] 0.75–0.82). The corresponding odds ratio was 0.78 (95% CI 0.69–0.88) in the sibling and 0.72 (95% CI 0.66–0.79) in the cousin comparison analysis.

**Conclusions:**

This nationwide, family-based study provides support for a protective effect of maternal smoking on offspring type 1 diabetes. Mechanistic studies are needed to elucidate the underlying pathways behind this link.

**Supplementary Information:**

The online version contains supplementary material available at 10.1186/s12916-022-02447-5.

## Background

Type 1 diabetes is one of the most common chronic diseases in childhood, and incidence has been increasing globally over the past three decades at an average annual rate of 3–4% [1]. Autoantibodies associated with the development of type 1 diabetes may appear already before the age of 6 months but most commonly during the second year of life [2]. This phenomenon indicates that early life factors may contribute to the development of type 1 diabetes, although few risk factors have been established [2].

Maternal smoking during pregnancy was reported to be associated with a reduced risk of type 1 diabetes in the offspring in a number of studies [3, 4], including large prospective studies from Sweden [5], Norway [6], Australia [3], and Finland [7]. Since these are observational studies, the results can be confounded because mothers who smoke during pregnancy may differ from other women in ways including child-rearing practices and genetic factors, which may affect the offspring’s risk of type 1 diabetes but were not adjusted for in most previous studies [3, 5, 7–17]. Factors that have been linked to an increased risk of type 1 diabetes in children, which might also differ between smoking and non-smoking mothers, include the early introduction of certain foods such as gluten and fruit and high intake of milk and carbohydrates during childhood [18], as well as childhood exposure to infections [1]. In addition, non-smoking mothers are more likely to breastfeed [19, 20], which has been reported to reduce the risk of type 1 diabetes in their children [18]. Since it is impossible to perform randomized clinical trials to assess smoking effects on fetal development, other designs are needed to minimize potential confounding.

Family-based designs, such as sibling and cousin comparisons, are quasi-experiments that allow us to control for factors shared by relatives [21]. For example, by comparing diabetes risk in siblings who are discordant for fetal exposure to smoking, we can reduce the confounding from genetic (full siblings share 50% of their segregating genes), maternal (intra-uterine and child-rearing), and childhood environmental factors. Cousins share fewer familial (genetic and environmental) factors than siblings; by combining cousin and sibling designs, we can therefore explore the degree to which genetic and environmental factors account for an observed association [21].

We aimed to assess the association between maternal smoking during pregnancy and offspring type 1 diabetes while accounting for familial confounding. To this aim, we used nationwide data from Swedish national registers and family-based designs. For comparison purposes, we also investigated the association of maternal smoking during pregnancy with offspring type 2 diabetes.

## Methods

### Registry linkage

**Fig. 1:**
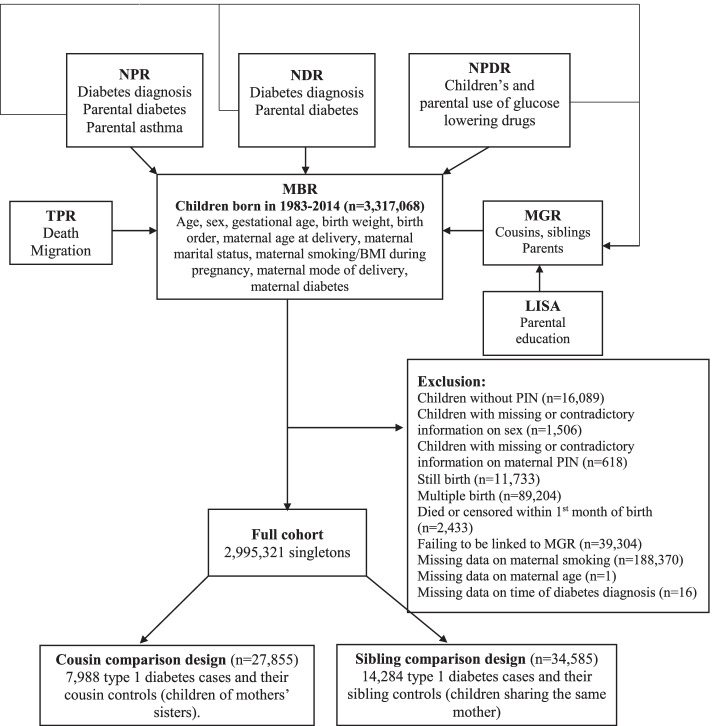
Flow chart of the family-based designs. MBR, Medical Birth Register; MGR, Multi-Generation Register; LISA, Longitudinal Integrated Database for Health Insurance and Labor Market Studies; TPR, Total Population Register; NPR, National Patient Register; NDR, National Diabetes Register; NPDR, National Prescribed Drug Register; PIN, personal identity number

Data for this study were retrieved from national registers in Sweden, including the Medical Birth Register (MBR) [22], the Multi-Generation Register (MGR) [23], the Longitudinal Integrated Database for Health Insurance and Labor Market Studies (LISA) [24], the National Patient Register (NPR) [25], the National Diabetes Register (NDR) [26], the National Prescribed Drug Register (NPDR) [27, 28], and the Total Population Register (TPR) [29]. These registers were linked by the unique personal identity number (PIN) assigned to every Swedish citizen (Fig. 1). The study was approved by the Swedish ethical review board (2021-02881).

### Study population

We identified all children born (*n* = 3,317,068) between January 1, 1983, and December 31, 2014 and their mothers through the MBR. We excluded children without PIN (*n* = 16,089), children with missing or contradictory information on sex (*n* = 1506), children with missing or contradictory information on maternal PIN (*n* = 618), stillbirths (*n* = 11,733), multiple births (*n* = 89,204), children who died or were censored within the first month of birth (*n* = 2433), individuals failing to be linked to the MGR for relatives’ information (*n* = 39,304), individuals with missing data on maternal smoking during early pregnancy (*n* = 188,370), maternal age at delivery (*n* = 1), or time of diabetes diagnosis (*n* = 16). A total of 321,747 (9.7%) children were excluded, leaving an analytical sample consisting of 2,995,321 children (Fig. 1).

### Smoking

Information on maternal smoking during pregnancy was retrieved from MBR, which contains self-reported smoking information since 1983 [22]. Trained midwives collected information on smoking from expectant women at their first antenatal visit (typically at 8–12 weeks of pregnancy) using standardized questionnaires. Available response options included non-smoking, 1–9 cigarettes per day, and ≥ 10 cigarettes per day [30]. A previous study showed that the validity of self-reported smoking information during early pregnancy is high (*κ* = 0.82 for agreement between self-reported smoking information and smoking status classified through cotinine measurements) in Sweden [31]. MBR also includes information on smoking at 30–32 weeks of pregnancy since 1990, but the missing rate was 75.2% in the 1990s, 24% in the 2000s, and 12.7% in the 2010s. Unless stated otherwise, maternal smoking during pregnancy refers to maternal smoking at the first antenatal visit in the following sections.

### Diabetes

The children were followed for a diagnosis of type 1 diabetes at age ≤ 18 years by linkage to NPR, NDR, and NPDR. NPR was established in 1964 and covers all inpatient care in Sweden since 1987 and outpatient specialist care provided by public and private caregivers since 2001 [25]. NPR coded diseases according to the International Classification of Diseases (ICD). NDR was created in 1996 and is the largest diabetes register in the world (www.ndr.nu) [26]. NPDR was established in July 2005 and includes all prescribed drugs dispensed at Swedish pharmacies, with prescribed drugs coded by the Anatomical Therapeutic Code (ATC) [27].

Type 1 diabetes was defined as receiving a diagnosis of type 1 diabetes at age ≤ 18 years (a) in NPR (ICD-8 code 250, ICD-9 code 250, ICD-10 code E10) or (b) in NDR or (c) the exclusive use of insulin at age ≤ 18 years recorded in NPDR. Patients were also defined as type 1 diabetes if they were diagnosed with diabetes of unknown type or of different types before age of 18 years in NPR or NDR and did not have a record of oral glucose-lowering drugs in NPDR.

We also identified cases of type 2 diabetes in these registers as a secondary outcome. Type 2 diabetes was defined as receiving a diagnosis of type 2 diabetes (a) in NPR (ICD-10 code E11) or (b) in NDR, with or without the use of glucose-lowering drugs. Patients were also defined as type 2 diabetes if they were diagnosed with diabetes of unknown type in NPR or NDR but were exclusively prescribed with non-insulin glucose-lowering drugs.

The date of diagnosis was defined according to the first recording in NDR, NPR, or NPDR, whichever came first. In addition, we checked the vital status and migration status of participants by linking to TPR [29].

### Covariates

In addition to basic characteristics such as age, sex, and year of birth, we considered all available perinatal factors as covariates to minimize confounding. Some of these factors (gestational age [32], birth weight for gestational age [32], maternal age at delivery [1], maternal body mass index [BMI] during early pregnancy [1], and parental history of diabetes [33]) have been reported to be associated with the risk of type 1 diabetes. MBR contains information on year and month of birth, sex, gestational age, birth weight, birth order, maternal age at delivery, maternal marital status, maternal BMI at the first antenatal visit, and mode of delivery. Data on the parental highest degree of education attained before childbirth were obtained from LISA [24]. Parental diabetes at childbirth was identified from NDR, NPR, and NPDR. Parental asthma at childbirth was identified from the NPR.

#### Family-based, nested case–control study

Within the study population, we linked siblings and cousins to each other through their parents and grandparents in MGR, which contains information on parents of children born from 1932 onwards [23]. In the sibling comparison design, children with type 1 diabetes were matched to their siblings (same mother), who were alive and free of diabetes at the age when the case was diagnosed [34]. In the cousin comparison design, we only included the offspring of sisters and matched all children with type 1 diabetes to their cousins who were alive and free of diabetes at the age when the case was diagnosed. Sibling (or cousin) groups that were discordant on both maternal smoking and type 1 diabetes diagnosis contributed to the estimates for maternal smoking during pregnancy [34, 35]. However, sibling (or cousin) groups concordant on maternal smoking were informative for the estimates for covariates and were therefore included as participants [34]. The same approach of matching was used for the cousin and sibling comparison analyses of type 2 diabetes.

### Statistical analysis

#### Cohort analysis

For the analysis of type 1 diabetes, follow-up time was calculated from the date of birth to the occurrence of diabetes, death, migration, December 31 of the year when children turned 18 years old, or December 31, 2020, whichever came first. Analyses were similar for type 2 diabetes but without censoring for age. Cox proportional hazards regression models estimated the hazard ratios (HRs) and 95% confidence intervals (CIs) for maternal smoking during pregnancy. The models were fitted with age as the time scale and a gamma-frailty component [36] to account for the familial clustering of participants. We inspected the proportional hazards assumption through a visual comparison of HRs for the first (attained age 1–9 years) and the second half of the time scale (attained age 10–18 years), and there was no obvious evidence of departure from this assumption.

#### Sibling and cousin comparison analyses

In the sibling (or cousin) comparison analysis, conditional logistic models estimated the odds ratios (ORs) and 95% CIs of type 1 diabetes and type 2 diabetes for maternal smoking during pregnancy, conditioning on sibling (or cousin) groups.

Models in the cohort and sibling and cousin analyses were all adjusted for sex, year of birth (1983–1984, 1985–1989, 1990–1994, 1995–1999, 2000–2004, 2005–2009, 2010–2014) (model 1), maternal and paternal education, maternal age at delivery, maternal BMI at the first antenatal visit, maternal diabetes status (any type of diabetes but not gestational diabetes) at childbirth, paternal diabetes (any type of diabetes) at childbirth, birth order (model 2), gestational age, and birth weight for gestational age (model 3). We tested for the potential difference in the incidence of type 1 diabetes between the two maternal smoking dose groups (1–9 cigarettes per day and ≥ 10 cigarettes per day) by treating the exposure as two dummy variables (*d*_1_, *d*_2_): *d*_1_ = 0 and *d*_2_ = 0 for non-smoking, *d*_1_ = 1 and *d*_2_ = 0 for 1–9 cigarettes per day, and *d*_1_ = 1 and *d*_2_ = 1 for ≥ 10 cigarettes per day. The *P* value for *d*_2_ indicated whether there was a difference in type 1 diabetes incidence between the two maternal smoking dose groups. Participants with missing values on categorical covariates were treated as a separate group in the analyses, and those with missing values on continuous covariates were assigned the median value. A binary variable was included in the analyses to indicate if values were imputed.

##### Timing of maternal smoking during pregnancy

We also explored the incidence of offspring type 1 diabetes in relation to maternal smoking only at the first antenatal visit, only at 30–32 weeks of pregnancy, and sustained smoking during pregnancy (both at the first antenatal visit and at 30–32 weeks of pregnancy) in the subset of children (*n* = 1,363,501) born after 2000 when such information was recorded with a relatively low missing rate.

#### Sensitivity analyses

We did subgroup analyses according to sex and birth year. To assess the generalizability of the sibling results, the cohort analyses were performed separately in individuals with and without siblings.

We also ran the analyses with additional adjustments for maternal marital status, mode of delivery (which was reported to be not associated with type 1 diabetes by a previous sibling comparison study [37]), and parental history of asthma (to account for parental asthma’s potential influence on maternal smoking and the shared genetic susceptibility between asthma and type 1 diabetes [38, 39]) and performed complete-case analysis where individuals with missing data on covariates were excluded. We additionally performed a sensitivity analysis by excluding children with parental history of diabetes at childbirth. We limited the sibling comparison analyses to full siblings and the cousin comparison analyses to full cousins (offspring of sisters who were full siblings). We also restricted the sibling (cousin) analyses to siblings (cousins) born within 5 years, who may share childhood environmental factors to a larger extent than those with a larger age difference. The sibling comparison design assumes no carryover effect; that is, the first sibling’s maternal smoking during pregnancy should not affect the subsequent sibling’s risk of type 1 diabetes [40–42]. The carryover effect is less of a concern in the cousin comparison design [42]. To further minimize the potential bias from a carryover effect, we limited the cousin comparison analysis to first-born cousins. To assess the potential for unmeasured confounding, we calculated an *E* value [43] (Additional file 1: eMethods) based on the OR of type 1 diabetes associated with maternal smoking from the sibling analysis (model 3). Finally, we calculated the proportion of the increasing incidence of type 1 diabetes that could be attributed to the decline in maternal smoking for children born between 1983 and 2004 (the end of follow-up was the same as the main analyses), a birth cohort period when the incidence of type 1 diabetes kept increasing. This was done by first calculating the HR of type 1 diabetes by birth year without (model 0) and with adjustment (model 1) for maternal smoking. To assess the proportion of the excess risk attributable to confounding by maternal smoking, we then calculated the percent difference between the crude and adjusted HR [44] ([HR_model0_ − HR _model1_]/[HR_model0_ − 1] × 100%), with adjustment for sex in both models.

The gamma-frailty models were performed in R 4.1.0, and other statistical analyses were performed in Stata 16.1 (StataCorp). All hypothesis tests were 2-sided.

## Results

### Characteristics

A total of 18,617 type 1 diabetes cases diagnosed at age ≤ 18 years occurred during a median follow-up of 18.2 years in the cohort of 2,995,321 individuals. The median age at type 1 diabetes diagnosis was 9.4 years. The cousin comparison included 7988 type 1 diabetes cases who had at least one (range 1–26; median 3) eligible cousin (*n* = 27,855), and the sibling analyses included 14,284 type 1 diabetes with at least one (range 1–10; median 1) eligible sibling (*n* = 34,585) (Fig. 1). Compared to children without type 1 diabetes, cases were more likely to be boys and have parents with diabetes (Table 1). The overall proportion of children exposed to maternal smoking during pregnancy was 15.7%. The proportion decreased with an increased year of birth (from 30.6% in 1983–1984 to 5.9% in 2010–2014), gestational age, birth weight for gestational age, maternal age, and parental educational levels. Single mothers were more likely to smoke during pregnancy than mothers living with their children’s fathers (Additional file 1: eTable 1).

**Table 1:** Characteristics of children by type 1 diabetes status in different study designs^a^

	Cohort analysis		Cousin analysis		Sibling analysis	
	No type 1 diabetes	Type 1 diabetes	Not any diabetes	Type 1 diabetes	Not any diabetes	Type 1 diabetes
Overall	2,976,704 (99.4)	18,617 (0.6)	21,036 (72.5)	7988 (27.5)	21,302 (59.9)	14,284 (40.1)
Boys	1,529,498 (51.4)	10,166 (54.6)	10,729 (51.0)	4399 (55.1)	10,908 (51.2)	7816 (54.7)
Year of birth						
1983–1984	164,968 (5.5)	888 (4.8)	1141 (5.4)	302 (3.8)	799 (3.8)	451 (3.2)
1985–1989	469,384 (15.8)	2811 (15.1)	3800 (18.1)	12,203 (15.1)	3537 (16.6)	2053 (14.4)
1990–1994	534,392 (18.0)	3892 (20.9)	4918 (23.4)	1849 (23.1)	5038 (23.7)	3236 (22.7)
1995–1999	407,468 (13.7)	3404 (18.3)	3769 (17.9)	1476 (18.5)	4192 (19.7)	2790 (19.5)
2000–2004	421,330 (14.2)	3533 (19.0)	3436 (16.3)	1531 (19.2)	3782 (17.8)	2842 (19.9)
2005–2009	468,632 (15.7)	2696 (14.5)	2624 (12.5)	1090 (13.6)	2656 (12.5)	2032 (14.2)
2010–2014	510,530 (17.2)	1393 (7.5)	1348 (6.4)	537 (6.7)	1298 (6.1)	80 (6.2)
Gestational age in days, mean (SD)	278.8 (12.2)	277.9 (12.2)	278.4 (12.4)	278.0 (12.0)	278.3 (12.0)	278.0 (12.0)
Birth weight for gestational age						
Small for gestational age	70,763 (2.4)	382 (2.1)	466 (2.2)	167 (2.1)	388 (1.8)	267 (1.9)
Normal	2,793,435 (93.8)	17,350 (93.2)	19,663 (93.5)	7466 (93.5)	19,783 (92.9)	13,316 (93.2)
Large for gestational age	103,661 (3.5)	834 (4.5)	850 (4.0)	337 (4.2)	1066 (5.0)	666 (4.7)
Unknown	8845 (0.3)	51 (0.3)	57 (0.3)	18 (0.2)	65 (0.3)	35 (0.2)
Birth order						
1	1,265,549 (42.5)	7851 (42.2)	8667 (41.2)	3273 (41.0)	7523 (35.3)	5203 (36.4)
2	1,082,395 (36.4)	6833 (36.7)	7545 (35.9)	2966 (37.1)	7617 (35.8)	5796 (40.6)
3 +	628,760 (21.1)	3933 (21.1)	4824 (22.9)	1749 (21.9)	6162 (28.9)	3285 (23.0)
Maternal age at delivery in years, mean (SD)	29.7 (5.2)	29.7 (5.1)	28.8 (5.1)	29.4 (5.1)	29.0 (5.1)	29.5 (5.0)
Maternal marital status						
Living with child’s father	2,751,651 (92.4)	17,282 (92.8)	19,354 (92.0)	7420 (92.9)	19,788 (92.9)	13,362 (93.5)
Single	56,402 (1.9)	330 (1.8)	452 (2.1)	151 (1.9)	347 (1.6)	227 (1.6)
Others	89,494 (3.0)	482 (2.6)	641 (3.0)	196 (2.5)	553 (2.6)	293 (2.1)
Unknown	79,157 (2.7)	523 (2.8)	589 (2.8)	221 (2.8)	614 (2.9)	402 (2.8)
Maternal educational level						
Pre-secondary	386,663 (13.0)	2343 (12.6)	2799 (13.3)	980 (12.3)	2705 (12.7)	1600 (11.2)
Upper-secondary	567,201 (19.1)	4343 (23.3)	5282 (25.1)	2105 (26.4)	5108 (24.0)	3459 (24.2)
High school	567,452 (19.1)	3573 (19.2)	3684 (17.5)	1495 (18.7)	3764 (17.7)	2736 (19.2)
Post-secondary or higher	914,211 (30.7)	5387 (28.9)	5018 (23.9)	2127 (26.6)	5547 (26.0)	4198 (29.4)
Unknown	541,177 (18.2)	2971 (16.0)	4253 (20.2)	1281 (16.0)	4178 (19.6)	2291 (16.0)
Paternal educational level						
Pre-secondary	492,235 (16.5)	2975 (16.0)	3597 (17.1)	1258 (15.7)	3213 (15.1)	2098 (14.7)
Upper-secondary	716,097 (24.1)	5451 (29.3)	62,200 (29.5)	2491 (31.2)	6296 (29.6)	4334 (30.3)
High school	547,304 (18.4)	3226 (17.3)	3287 (15.6)	1344 (16.8)	3312 (15.5)	2372 (16.6)
Post-secondary or higher	785,763 (26.4)	4607 (24.7)	4347 (20.7)	1828 (22.9)	5002 (23.5)	3645 (25.5)
Unknown	435,305 (14.6)	2358 (12.7)	3605 (17.1)	1067 (13.4)	3479 (16.3)	1835 (12.8)
Maternal BMI in kg/m^2^ during pregnancy^b^, mean (SD)	23.9 (4.3)	24.1 (4.3)	23.9 (4.3)	24.2 (4.3)	24.1 (4.5)	24.1 (4.3)
Maternal cesarean section at delivery	399,662 (13.4)	2724 (14.6)	2595 (12.3)	1107 (13.9)	2576 (12.1)	1890 (13.2)
Maternal diabetes at childbirth^c^	24,923 (0.8)	593 (3.2)	340 (1.6)	256 (3.2)	560 (2.6)	392 (2.7)
Paternal diabetes at childbirth	21,731 (0.7)	865 (4.6)	123 (0.6)	371 (4.6)	760 (3.6)	623 (4.4)
Maternal asthma at childbirth	158,268 (5.3)	1085 (5.8)	1093 (5.2)	475 (5.9)	1141 (5.4)	815 (5.7)
Paternal asthma at childbirth	35,258 (1.2)	237 (1.3)	202 (1.0)	114 (1.4)	259 (1.2)	171 (1.2)

*SD* standard deviation, *BMI* body mass index

^a^Values are presented as numbers (proportions [%]) unless stated otherwise

^b^At first antenatal visit, mostly during 8–12 weeks of pregnancy

^c^Any type of diabetes but not gestational diabetes

### Maternal smoking during pregnancy and offspring type 1 diabetes

**Fig. 2:**
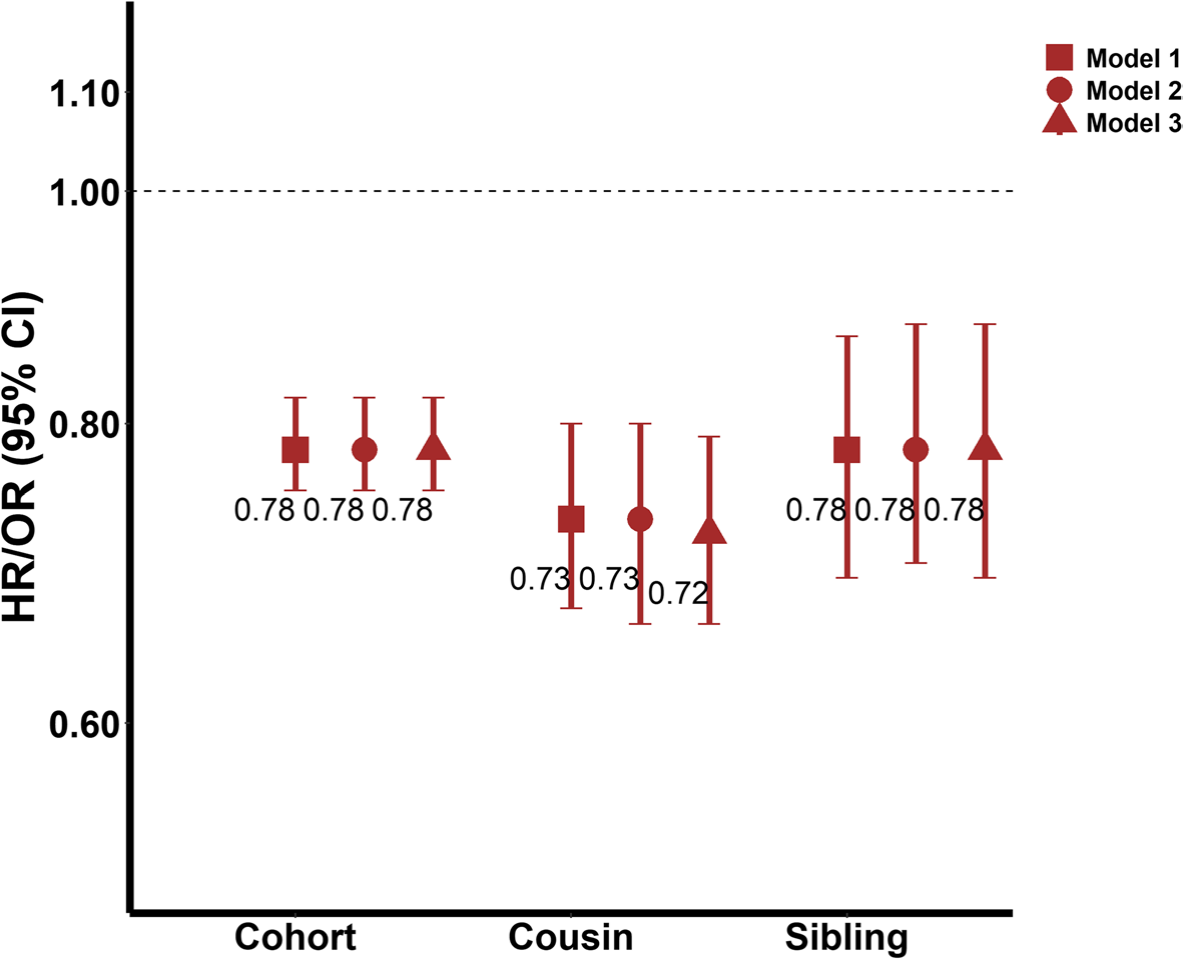
HRs/ORs (95% CIs) of type 1 diabetes for maternal smoking versus non-smoking during pregnancy in different study designs. HR, hazard ratio; OR, odds ratio; CI, confidence interval. Model 1 was adjusted for sex and year of birth. The model in the cohort analysis was fitted with a frailty component to take into account the non-independence among children born by the same mother. The model in the cousin (or sibling) analysis was conditioning on cousin (or sibling) groups. Model 2 was additionally adjusted for maternal education, paternal education, maternal age at delivery, maternal body mass index during pregnancy, maternal diabetes, paternal diabetes, and birth order on the basis of model 1. Model 3 was additionally adjusted for gestational age and birth weight for gestational age on the basis of model 2

In the cohort analysis, the HR (95% CI) of offspring type 1 diabetes was 0.78 (0.75–0.82) for maternal smoking versus non-smoking during pregnancy (Fig. 2). The inverse association was similar in the cousin (OR 0.72, 95% CI 0.66–0.79) and the sibling comparison analysis (OR 0.78, 95% CI 0.69–0.88) (Fig. 2). Moreover, the associations were consistent across the three models.

Both smoking 1–9 cigarettes/day and ≥ 10 cigarettes/day were associated with a reduced risk of type 1 diabetes in the offspring (Table 2), with an OR of 0.80 (95% CI 0.71–0.90) and 0.71 (95% CI 0.60–0.84), respectively, in the sibling analyses (Table 2).

**Table 2:** Associations^a^ between maternal smoking during pregnancy and offspring type 1 diabetes in different study designs

Smoking status	Non-smoking	1–9 cigarettes/day	≥ 10 cigarettes/day	*P*^†^
Cohort analysis				
No. of cases	16,084	1684	849	
Person years	38,819,013	5,235,610	2,863,183	
Incidence (no./10,000 person years)	4.1	3.2	3.0	
Model 1	1.00	0.80 (0.76–0.85)	0.75 (0.70–0.80)	0.100
Model 2	1.00	0.80 (0.76–0.84)	0.74 (0.69–0.80)	0.076
Model 3	1.00	0.80 (0.76–0.85)	0.74 (0.69–0.80)	0.066
Cousin comparison analysis				
No. of cases	6765	801	422	
Model 1	1.00	0.75 (0.68–0.83)	0.69 (0.60–0.79)	0.245
Model 2	1.00	0.75 (0.68–0.83)	0.67 (0.58–0.77)	0.114
Model 3	1.00	0.75 (0.68–0.83)	0.66 (0.58–0.76)	0.111
Sibling comparison analysis				
No. of cases	12,483	1193	608	
Model 1	1.00	0.79 (0.71–0.89)	0.70 (0.60–0.83)	0.116
Model 2	1.00	0.80 (0.71–0.90)	0.71 (0.60–0.84)	0.119
Model 3	1.00	0.80 (0.71–0.90)	0.71 (0.60–0.84)	0.115

Model 1 was adjusted for sex and year of birth. The Cox model in the cohort analysis was fitted with a gamma-frailty component to account for the clustering of siblings from the same mother. The conditional logistic model in the sibling (or cousin) comparison analysis conditioned on sibling (or cousin) groups

Model 2 was additionally adjusted for maternal education, paternal education, maternal age at delivery, maternal body mass index during pregnancy, maternal history of diabetes, paternal history of diabetes, and birth order based on model 1

Model 3 was additionally adjusted for gestational age and birth weight for gestational age based on model 2

^†^The *P* value indicted whether there was a difference in the incidence of type 1 diabetes between maternal smoking 1–9 cigarettes/day and ≥ 10 cigarettes/day

^a^Hazard ratios (95% confidence intervals) are presented for the cohort analysis, and odds ratios (95% confidence intervals) are presented for the cousin and sibling comparison analyses

In the subset born from 2000 onwards with information on the timing of smoking, cohort analyses revealed a reduced risk primarily in children of mothers who smoked persistently throughout the pregnancy (HR 0.83, 95% CI 0.74–0.93). In sibling analyses, numbers were small and the risk reduction appeared similar across the exposure windows, i.e., OR (95% CI) was 0.81 (0.55–1.19), 0.66 (0.36–1.21), and 0.74 (0.48–1.13) respectively for smoking only at the first antenatal visit, only at 30–32 weeks of pregnancy, and sustained throughout the pregnancy (Additional file 1: eTable 2).

### Maternal smoking during pregnancy and offspring type 2 diabetes

A total of 3477 type 2 diabetes cases (median age at diagnosis 26.5 years) occurred during a median follow-up of 21.5 years. There was a positive association between maternal smoking and type 2 diabetes in the cohort analysis (HR 1.90, 95% CI 1.77–2.04) which was attenuated in the cousin comparison analysis (OR 1.43, 95% CI 1.18–1.73) and even more so in the sibling analysis (OR 1.23, 95% CI 0.93–1.62) (Additional file 1: eFig. 1).

### Sensitivity analyses

The association between maternal smoking during pregnancy and offspring type 1 diabetes was similar when cohort analyses were performed separately in participants with and without siblings (Additional file 1: eTable 3). Sex and year of birth did not seem to modify the associations in neither cohort (Additional file 1: eTable 3) nor cousin or sibling comparison analyses (Additional file 1: eTable 4). The results were similar after additional adjustment for maternal marital status, mode of delivery, or parental asthma [38, 39] at childbirth, by the exclusion of individuals with missing data on any covariate and by the exclusion of children with maternal or paternal diabetes at childbirth (Additional file 1: eTables 5–7). Similarly, associations remained in cousin analyses restricted to full cousins, cousins born within 5 years, and first-born cousins (Additional file 1: eTable 6), as well as in sibling analyses restricted to full siblings and siblings within 5 years of age difference (Additional file 1: eTable 7). The *E* value was 1.88 (1/OR + sqrt[1/OR × (1/OR − 1)] = 1/0.78 + sqrt[1/0.78 × (1/0.78 − 1)]) calculated based on the estimated OR in model 3 of the sibling comparison analysis, which controlled for the most unmeasured familial confounders. The incidence of type 1 diabetes rose gradually for children born from 1983 to 2004 and the HR per birth year was estimated at 1.0267 (95% CI 1.0238–1.0295). After adjustment for maternal smoking during pregnancy, whose prevalence decreased from 30.6% in 1983–1984 to 10.3% in 2000–2004, the corresponding HR was 1.0239 (95% CI 1.0210–1.0268), and we estimated that 10% of the 2.67% annual increase in the incidence of type 1 diabetes for children born between 1983 and 2004 could be attributed to a decline in maternal smoking during pregnancy.

## Discussion

### Main findings

This nationwide prospective study shows that children exposed to maternal smoking during pregnancy have a 22% lower risk of developing type 1 diabetes during childhood compared to their unexposed siblings. This confirms the results of previous observational studies [3, 45] and suggests that maternal smoking during pregnancy has the potential to prevent offspring type 1 diabetes.

### Main findings in relation to previous studies

Previous studies on the link between maternal smoking during pregnancy and offspring type 1 diabetes include 15 cohort and 13 case–control studies based on European, US, and Australian populations [4]. We addressed this association using cohort, sibling, and cousin designs, the latter two of which allowed us to control for potential unmeasured confounders shared within families. The observed association between maternal smoking during pregnancy and offspring type 1 diabetes was consistent across the three designs, and the effect size was in line with the RR of 0.78, observed in a recent meta-analysis based on 22 studies [4]. It was also consistent with the pooled RR of 0.72 reported in another meta-analysis based on five population-based prospective studies [3]. This indicates that the reduced risk of type 1 diabetes seen in children prenatally exposed to smoking is unlikely to be explained by confounding and, furthermore, that the influence of familial (genetic and early environmental) factors is minor. According to our estimated *E* value, an uncontrolled confounder, which should be common in the population [43], needs to have a risk ratio of 1.88 with both the exposure and outcome to fully explain away the inverse association between maternal smoking during pregnancy and offspring type 1 diabetes. An Australian cohort study reported an *E* value of 1.67 for the observed point estimate adjusted for parental basic characteristics, parity, pre-pregnancy hypertension and diabetes, and birth year [3]. Compared to the Australian study, we had the opportunity to adjust for both unmeasured familial factors and a range of measured perinatal factors including mode of delivery, maternal BMI, parental diabetes, and gestational age, and we are not aware of any other factor with such a strong association to both maternal smoking and type 1 diabetes.

A few prospective studies have assessed the timing of prenatal exposure to smoking and the reduced risk of type 1 diabetes was primarily observed in the offspring of mothers who smoked throughout the pregnancy [3, 6, 7]. Our cohort-based results were in line with these observations whereas the results of the sibling analyses, although based on small numbers were consistent with a reduced risk also in the offspring of women who quit smoking after the first trimester.

There was a positive association between offspring type 2 diabetes and maternal smoking during pregnancy in the cohort analyses, which was attenuated in the sibling comparison analysis. This suggests that familial confounding contributes to the association observed in the full cohort. These findings are in line with those of a previous meta-analysis of five studies [46] showing no association between maternal smoking during pregnancy and offspring type 2 diabetes.

### Potential mechanisms

The inverse association between maternal smoking during pregnancy and offspring type 1 diabetes could hypothetically be attributed to the immunosuppressive effects of nicotine. Experimental studies show that nicotine can activate nicotinic acetylcholine receptors (nAChRs) in T cells [47, 48]. The activation of nAChRs may subsequently suppress systemic inflammation and autoimmunity [47, 48]. Nicotine-induced immunosuppression is associated with preserved insulin content and reduced incidence of diabetes in type 1 diabetes-prone mice models [49]. Experimental studies indicate that the immunosuppressive and anti-inflammatory effects of prenatal smoking exposure remain also postnatally [50, 51]. The hypothesis of immunosuppression is partly supported by the fact that autoantibodies of type 1 diabetes mostly appear within the first 2 years after birth, close to the time of fetal exposure [2]. In this context, it is noteworthy that parental smoking during childhood appears unrelated to offspring type 1 diabetes risk [6], supporting that the fetal period may be a sensitive period for a proposed smoking effect. The lack of association observed between maternal smoking and type 2 diabetes supports that the mechanism may involve effects related primarily to autoimmunity rather than other diabetogenic processes. Future mechanistic studies are however warranted to elucidate the potential pathways linking maternal smoking to offspring risk of type 1 diabetes. While these results do not have immediate clinical relevance, they do provide insights into the etiology and pathogenesis of type 1 diabetes.

### Strength and limitations

Strengths include the use of nationwide data, with almost 3 million children of whom 18,617 developed type 1 diabetes. Follow-up was made through the combination of national patient, drug, and diabetes registers to ensure a high coverage of diabetes cases and virtually no loss to follow-up. The quality of the registers is high [25, 26]; a diagnosis of type 1 diabetes based on the NDR has been shown to be accurate in 97% of cases diagnosed at age ≤ 30 [26]. A particular strength is the integration of a traditional cohort design with family-based designs of sibling and cousin comparison analyses; this allows us to control for unmeasured environmental and genetic confounders shared within families. In addition, we could adjust for a range of perinatal factors based on information recorded at birth. The possibility to compare the results for type 1 diabetes vs. type 2 diabetes provides clues to the potential mechanism linking maternal smoking to offspring type 1 diabetes. Limitations include self-reported information on maternal smoking during pregnancy which may be underreported due to social desirability. In this context, it is noteworthy that a previous study found an inverse association between offspring type 1 diabetes and levels of cotinine, a valid biomarker of nicotine exposure, measured in the cord blood [6]. Because ours was a prospective study, we can assume misclassification to be non-differential which leads to dilution rather than overestimation of associations. Furthermore, a previous Swedish study indicated that the validity of information on maternal smoking during early pregnancy is high [52]. We lacked information on breastfeeding, which has been linked to a reduced risk of offspring type 1 diabetes [18]. Such potential confounding is most likely reduced by comparing siblings. It should also be noted that since mothers who smoke can be expected to breastfeed less, failure to adjust for breastfeeding is unlikely to explain the inverse association between smoking and type 1 diabetes. Finally, we lacked information on paternal smoking during pregnancy and parental smoking during childhood. Still, a previous large cohort study did not observe an association between either parental smoking during childhood or paternal smoking during maternal pregnancy and offspring type 1 diabetes [6]. Regarding generalizability, it is noteworthy that the current literature on the association between maternal smoking and type 1 diabetes is based on Western populations, and whether the findings are generalizable to other ethnic groups remains to be investigated.

## Conclusions

This study provides evidence from family-based designs of sibling and cousin comparison analyses that maternal smoking during pregnancy may have a protective effect on offspring type 1 diabetes, adding evidence to the current knowledge on the development of type 1 diabetes. Despite these findings, smoking during pregnancy should be strongly advised against since it has several severe harmful effects on fetal and childhood health [35, 52, 53].

## Supplementary Information

**Additional file 1: eMethods.** The calculation of an E value. **eTable 1.** Maternal smoking status during pregnancy by levels of covariates in the full cohort. **eTable 2.** Timing of maternal smoking during pregnancy and offspring type 1 diabetes in different study designs. **eFigure 1.** HRs/ORs (95% CIs) of type 2 diabetes for maternal smoking versus nonsmoking during pregnancy in different study designs. **eTable 3.** Risk of offspring type 1 diabetes for maternal smoking versus nonsmoking during pregnancy by subgroups: cohort analysis. **eTable 4.** Risk of offspring type 1 diabetes for maternal smoking versus nonsmoking during pregnancy by subgroups: cousin and sibling analysis. **eTable 5.** Sensitivity analyses of type 1 diabetes risk in the offspring for maternal smoking versus nonsmoking during pregnancy: cohort analysis. **eTable 6.** Sensitivity analyses of type 1 diabetes risk in the offspring for maternal smoking versus nonsmoking during pregnancy: cousin analysis. **eTable 7.** Sensitivity analyses of type 1 diabetes risk in the offspring for maternal smoking versus nonsmoking during pregnancy: sibling analysis.

## Data Availability

The data that support the findings of this study are available from Statistics Sweden and the Swedish National Board of Health and Welfare, but restrictions apply to the availability of these data, which were used under license for the current study, and so are not publicly available.

## Abbreviations

ATC: Anatomical Therapeutic Code
BMI: Body mass index
CI: Confidence interval
HR: Hazard ratio
ICD: International Classification of Diseases
LISA: Longitudinal Integrated Database for Health Insurance and Labor Market Studies
MBR: Medical Birth Register
MGR: Multi-Generation Register
nAChRs: Nicotinic acetylcholine receptors
NDR: National Diabetes Register
NPDR: National Prescribed Drug Register
NPR: National Patient Register
OR: Odds ratio
PIN: Personal identity number
SD: Standard deviation
TPR: Total Population Register
