# Supplementary material for: Maternal smoking during pregnancy and type 1 diabetes in the offspring: a nationwide register-based study with family-based designs
Source: BMC Med. 2022 Aug 12;20:240. doi: 10.1186/s12916-022-02447-5 (PMC9373415; doi:10.1186/s12916-022-02447-5)
Supplement: Supplementary file 1 — Additional file 1: eMethods. The calculation of an E value. eTable 1. Maternal smoking status during pregnancy by levels of covariates in the full cohort. eTable 2. Timing of maternal smoking during pregnancy and offspring type 1 diabetes in different study designs. eFigure 1. HRs/ORs (95% CIs) of type 2 diabetes for maternal smoking versus nonsmoking during pregnancy in different study designs. eTable 3. Risk of offspring type 1 diabetes for maternal smoking versus nonsmoking during pregnancy by subgroups: cohort analysis. eTable 4. Risk of offspring type 1 diabetes for maternal smoking versus nonsmoking during pregnancy by subgroups: cousin and sibling analysis. eTable 5. Sensitivity analyses of type 1 diabetes risk in the offspring for maternal smoking versus nonsmoking during pregnancy: cohort analysis. eTable 6. Sensitivity analyses of type 1 diabetes risk in the offspring for maternal smoking versus nonsmoking during pregnancy: cousin analysis. eTable 7. Sensitivity analyses of type 1 diabetes risk in the offspring for maternal smoking versus nonsmoking during pregnancy: sibling analysis. [file 12916_2022_2447_MOESM1_ESM.docx]

Additional file 1: eMethods, etables 1-7, eFigure 1

eMethods. The calculation of an E value

eTable 1. Maternal smoking status during pregnancy by levels of covariates in the full cohort

eTable 2. Timing of maternal smoking during pregnancy and offspring type 1 diabetes in different study designs

eFigure 1. HRs/ORs (95% CIs) of type 2 diabetes for maternal smoking versus nonsmoking during pregnancy in different study designs

eTable 3. Risk of offspring type 1 diabetes for maternal smoking versus nonsmoking during pregnancy by subgroups: cohort analysis

eTable 4. Risk of offspring type 1 diabetes for maternal smoking versus nonsmoking during pregnancy by subgroups: cousin and sibling analysis

eTable 5. Sensitivity analyses of type 1 diabetes risk in the offspring for maternal smoking versus nonsmoking during pregnancy: cohort analysis

eTable 6. Sensitivity analyses of type 1 diabetes risk in the offspring for maternal smoking versus nonsmoking during pregnancy: cousin analysis

eTable 7. Sensitivity analyses of type 1 diabetes risk in the offspring for maternal smoking versus nonsmoking during pregnancy: sibling analysis

eMethods. The calculation of an E value

The E value represents the minimum strength of association, on the risk ratio scale, that an uncontrolled confounder would need to have with both maternal smoking and offspring type 1 diabetes to fully explain away the observed maternal smoking-type 1 diabetes association. E-value equals to OR + sqrt[OR × (OR − 1)] when OR>1 and equals to OR* + sqrt[OR* × (OR* − 1)] when OR<1 (OR* = 1/OR).

eTable 1. Maternal smoking status during pregnancy by levels of covariates in the full cohort ^a^

|  | **Nonsmoking** | **Maternal smoking during pregnancy** |
| --- | --- | --- |
| Overall | 2,524,655 (84.3) | 470,666 (15.7) |
| Sex | | |
| Boys | 1,296,932 (84.2) | 242,732 (15.8) |
| Girls | 1,227,723 (84.3) | 227,934 (15.7) |
| Year of birth | | |
| 1983-1984 | 115,056 (69.4) | 50,800 (30.6) |
| 1985-1989 | 340,310 (72.1) | 131,885 (27.9) |
| 1990-1994 | 418,988 (77.8) | 119,296 (22.2) |
| 1995-1999 | 350,338 (85.3) | 60,534 (14.7) |
| 2000-2004 | 381,097 (89.7) | 43,766 (10.3) |
| 2005-2009 | 437,113 (92.7) | 34,215 (7.3) |
| 2010-2014 | 481,753 (94.1) | 30,170 (5.9) |
| Gestational age in weeks | | |
| Very preterm: 22–32 | 17,300 (77.6) | 5,005 (22.4) |
| Preterm: 33–36 | 95,191 (80.0) | 23,791 (20.0) |
| Early term: 37–38 | 457,033 (82.6) | 96,112 (17.4) |
| Term: 39–40 | 1,310,620 (84.7) | 236,883 (15.3) |
| Postterm: 41+ | 643,795 (85.6) | 108,513 (14.4) |
| Unknown | 716 (66.4) | 362 (33.6) |
| Birth weight for gestational age | | |
| Small for gestational age | 50,069 (70.4) | 21,076 (29.6) |
| Normal | 2,372,383 (84.4) | 438,402 (15.6) |
| Large for gestational age | 95,194 (91.1) | 9,301 (8.9) |
| Unknown | 7,009 (78.8) | 1,887 (21.2) |
| Birth order | | |
| 1 | 1,080,712 (84.9) | 192,688 (15.1) |
| 2 | 934,043 (85.8) | 155,185 (14.2) |
| 3+ | 509,900 (80.6) | 122,793 (19.4) |
| Maternal age in years | | |
| <20 | 38,647 (63.2) | 22,524 (36.8) |
| 20-24 | 389,615 (76.0) | 123,183 (24.0) |
| 25-29 | 858,663 (84.6) | 155,979 (15.4) |
| 30-34 | 808,588 (88.0) | 110,074 (12.0) |
| 35-39 | 358,952 (88.0) | 48,816 (12.0) |
| 40+ | 70,190 (87.4) | 10,090 (12.6) |
| Maternal marital status | | |
| Living with child's father | 2,367,701 (85.5) | 401,232 (14.5) |
| Single | 33,515 (59.1) | 23,217 (40.9) |
| Other | 60,637 (67.4) | 29,339 (32.6) |
| Unknown | 62,802 (78.8) | 16,878 (21.2) |
| Maternal educational level | | |
| Pre-secondary | 270,605 (69.6) | 118,401 (30.4) |
| Upper-secondary | 451,625 (79.0) | 119,919 (21.0) |
| High school | 518,739 (90.8) | 52,286 (9.2) |
| Post-secondary or higher | 891,488 (96.9) | 28,110 (3.1) |
| Unknown | 392,198 (72.1) | 151,950 (27.9) |
| Paternal educational level | | |
| Pre-secondary | 368,832 (74.5) | 126,378 (25.5) |
| Upper-secondary | 589,473 (81.7) | 132,075 (18.3) |
| High school | 496,077 (90.1) | 54,453 (9.9) |
| Post-secondary or higher | 759,163 (96.1) | 31,207 (3.9) |
| Unknown | 311,110 (71.1) | 126,553 (28.9) |
| Maternal BMI during pregnancy ^b^ | | |
| Underweight | 66,664 (75.7) | 21,409 (24.3) |
| Normal | 1,385,045 (86.1) | 223,954 (13.9) |
| Overweight | 470,000 (86.8) | 71,740 (13.2) |
| Obese | 185,107 (85.2) | 32,187 (14.8) |
| Unknown | 417,839 (77.5) | 121,376 (22.5) |
| Maternal caesarean section at delivery | | |
| No | 2,182,653 (84.2) | 410,282 (15.8) |
| Yes | 342,002 (85.0) | 60,384 (15.0) |
| Maternal diabetes at childbirth | | |
| No | 2,503,118 (84.3) | 466,687 (15.7) |
| Yes | 21,537 (84.4) | 3,979 (15.6) |
| Paternal diabetes at childbirth | | |
| No | 2,505,190 (84.3) | 467,535 (15.7) |
| Yes | 19,465 (86.1) | 3,131 (13.9) |
| Maternal asthma at childbirth | | |
| No | 2,387,280 (84.2) | 448,688 (15.8) |
| Yes | 137,375 (86.2) | 21,978 (13.8) |
| Paternal asthma at childbirth | | |
| No | 2,493,615 (84.2) | 466,211 (15.8) |
| Yes | 31,040 (87.4) | 4,455 (12.6) |

BMI: body mass index.

^a^ Values are presented as numbers (proportions [%]).

^b^ At first antenatal visit, mostly during 8-12 weeks’ of pregnancy.

eTable 2. Timing of maternal smoking during pregnancy and offspring type 1 diabetes in different study designs

|  | **Never smoking** | **Only at first antenatal visit** | **Only at 30-32 weeks of pregnancy** | **Sustained smoking ^a^** |
| --- | --- | --- | --- | --- |
| Cohort analysis |  |  |  |  |
| No. of cases | 6,850 | 143 | 52 | 312 |
| Person years | 15,972,446 | 332,364 | 133,308 | 881,869 |
| Incidence (No./10,000 person years) | 6.3 | 30.1 | 7.5 | 11.3 |
| HR (95% CI) | 1.00 | 1.00 (0.85-1.19) | 0.92 (0.70-1.21) | 0.83 (0.74-0.93) |
| Cousin analysis |  |  |  |  |
| No. of cases | 2,006 | 39 | 23 | 115 |
| OR (95% CI) | 1.00 | 0.78 (0.51-1.21) | 1.09 (0.61-1.93) | 0.83 (0.62-1.11) |
| Sibling analysis |  |  |  |  |
| No. of cases | 4,142 | 64 | 22 | 135 |
| OR (95% CI) | 1.00 | 0.81 (0.55-1.19) | 0.66 (0.36-1.21) | 0.74 (0.48-1.13) |

^a^ Smoking at both first antenatal visit (8-12 weeks of pregnancy) and at 30-32 weeks of pregnancy.

Models were adjusted for sex, year of birth, maternal education, paternal education, maternal age at delivery, maternal body mass index during pregnancy, maternal history of diabetes, paternal history of diabetes, birth order, gestational age and birth weight for gestational age. The model in the cohort analysis was fitted with a gamma-frailty component to account for the clustering of children born by the same mother. The cousin (or sibling) analysis was conditioning on cousin (or sibling) groups.


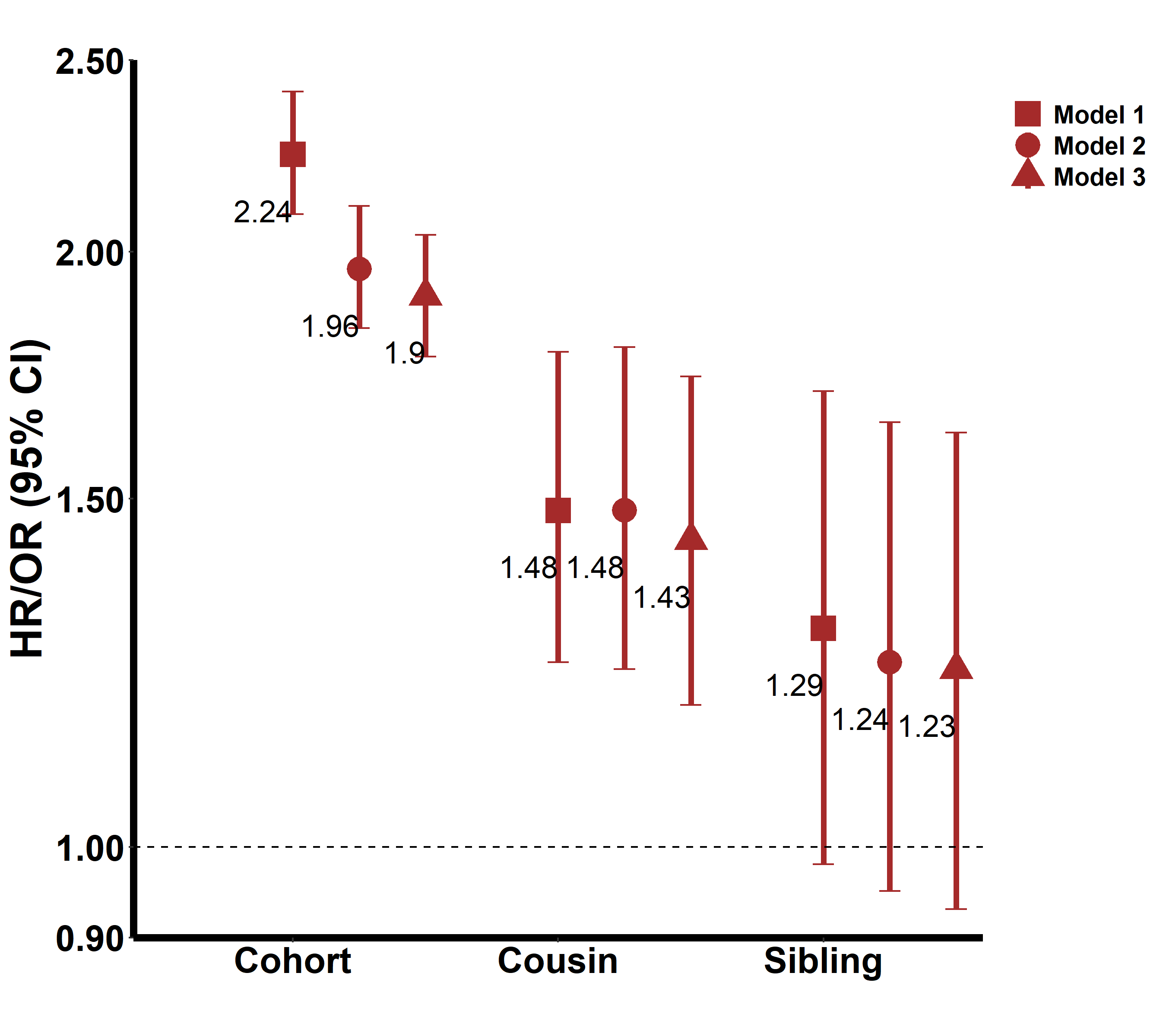


eFigure 1. HRs/ORs (95% CIs) of type 2 diabetes for maternal smoking versus nonsmoking during pregnancy in different study designs

HR: hazard ratio; OR: odds ratio; CI: confidence interval

Model 1 was adjusted for sex and year of birth. The model in the cohort analysis was fitted with a gamma-frailty component to account for the clustering of children born by the same mother. The model in the cousin (or sibling) analysis was conditioning on cousin (or sibling) groups.

Model 2 was additionally adjusted for maternal education, paternal education, maternal age at delivery, maternal body mass index during pregnancy, maternal diabetes, paternal diabetes, and birth order on the basis of model 1.

Model 3 was additionally adjusted for gestational age and birth weight for gestational age on the basis of model 2.

eTable 3. Risk of offspring type 1 diabetes for maternal smoking versus nonsmoking during pregnancy by subgroups: cohort analysis

|  | **Subgroups** | **No. of cases** | **HR (95% CI)** |
| --- | --- | --- | --- |
| **Sibling status** | Without siblings | 1,704 | 0.82 (0.71-0.93) |
|  | With siblings | 16,913 | 0.78 (0.74-0.82) |
| **Sex** | Boys | 10,166 | 0.80 (0.75-0.85) |
|  | Girls | 8,451 | 0.77 (0.72-0.82) |
| **Year of birth** | before 2000 | 10,995 | 0.77 (0.73-0.81) |
|  | 2000 onwards | 7,622 | 0.82 (0.74-0.90) |

OR: odds ratio; CI: confidence interval

Models fitted with a gamma-frailty to account for the clustering of children born by the same mother. They were adjusted for sex (when appropriate), year of birth, maternal education, paternal education, maternal age at delivery, maternal body mass index during pregnancy, maternal history of diabetes, paternal history of diabetes, birth order, gestational age and birth weight for gestational age.

eTable 4. Risk of offspring type 1 diabetes for maternal smoking versus nonsmoking during pregnancy by subgroups: cousin and sibling analysis

|  | **Subgroups** | **Cousin analysis** | |  | **Sibling analysis** | |
| --- | --- | --- | --- | --- | --- | --- |
|  |  | **No. of cases** | **OR (95% CI)** |  | **No. of cases** | **OR (95% CI)** |
| Sex | Boys | 3,311 | 0.72 (0.63-0.83) |  | 4,750 | 0.81 (0.66-1.00) |
|  | Girls | 2,665 | 0.85 (0.72-1.00) |  | 3,789 | 0.84 (0.67-1.06) |
|  | *P* for interaction | 0.668 | |  | 0.948 | |
| Year of birth | Before 2000 | 4,158 | 0.71 (0.64-0.80) |  | 7,794 | 0.78 (0.67-0.90) |
|  | 2000 onwards | 2,310 | 0.76 (0.60-0.97) |  | 4,584 | 0.82 (0.62-1.10) |
|  | *P* for interaction | 0.585 | |  | 0.541 | |

OR: odds ratio; CI: confidence interval

Models were conditioning on cousin (or sibling) groups, and were adjusted for sex (when appropriate), year of birth, maternal education, paternal education, maternal age at delivery, maternal body mass index during pregnancy, maternal history of diabetes, paternal history of diabetes, birth order, gestational age and birth weight for gestational age.

eTable 5. Sensitivity analyses of type 1 diabetes risk in the offspring for maternal smoking versus nonsmoking during pregnancy: cohort analysis

| **Sensitivity analyses** | **No. of cases** | **Incidence (no./10,000 person years)** | **HR (95% CI)** |
| --- | --- | --- | --- |
| Additionally adjusted for maternal marital status | 18,617 | 4.0 | 0.78 (0.75-0.82) |
| Additionally adjusted for mode of delivery | 18,617 | 4.0 | 0.78 (0.75-0.82) |
| Additionally adjusted for parental asthma ^a^ | 18,617 | 4.0 | 0.78 (0.75-0.82) |
| Complete-case analysis ^b^ | 12,526 | 4.3 | 0.81 (0.76-0.86) |
| Only among children without parental diabetes at childbirth | 17,198 | 3.7 | 0.78 (0.74-0.82) |

HR: hazard ratio; CI: confidence interval

Models were fitted with a gamma-frailty component to account for the clustering of children born by the same mother, and were adjusted for sex, year of birth, maternal education, paternal education, maternal age at delivery, maternal body mass index during pregnancy, maternal history of diabetes (when appropriate), paternal history of diabetes (when appropriate), birth order, gestational age and birth weight for gestational age. Models were additionally adjusted for maternal marital status, mode of delivery and parental asthma, when appropriate.

^a^ Parental history of asthma might affect the possibility of maternal smoking during pregnancy, and asthma shares some genetic susceptibility with type 1 diabetes.

^b^ Participants with missing information on maternal level of education, paternal level of education, maternal body mass index, gestational age, or birth weight were excluded from the analysis.

eTable 6. Sensitivity analyses of the risk of type 1 diabetes in the offspring for maternal smoking versus nonsmoking during pregnancy: cousin analysis

| **Sensitivity analyses** | **No. of cases** | **OR (95% CI)** |
| --- | --- | --- |
| Additionally adjusted for maternal marital status | 7,988 | 0.73 (0.66-0.80) |
| Additionally adjusted for mode of delivery | 7,988 | 0.72 (0.66-0.79) |
| Additionally adjusted for parental asthma | 7,988 | 0.72 (0.66-0.79) |
| Complete-case analysis | 4,493 | 0.76 (0.66-0.87) |
| Only among children without parental diabetes at childbirth | 7,269 | 0.72 (0.66-0.79) |
| Full cousins | 6,663 | 0.77 (0.69-0.85) |
| First-born cousins | 3,381 | 0.74 (0.64-0.86) |
| Only among cousins within 5 years of birth year gap | 5,684 | 0.78 (0.70-0.87) |

OR: odds ratio; CI: confidence interval

Models were conditioning on cousin groups, and were adjusted for maternal education, paternal education, maternal age at delivery, maternal body mass index during pregnancy, maternal history of diabetes (when appropriate), paternal history of diabetes (when appropriate), birth order, gestational age and birth weight for gestational age. Models were additionally adjusted for maternal marital status, mode of delivery and parental asthma, when appropriate.

eTable 7. Sensitivity analyses of the risk of type 1 diabetes in the offspring for maternal smoking versus nonsmoking during pregnancy: sibling analysis

| **Sensitivity analyses** | **No. of cases** | **OR (95% CI)** |
| --- | --- | --- |
| Additionally adjusted for maternal marital status | 14,284 | 0.78 (0.69-0.87) |
| Additionally adjusted for mode of delivery | 14,284 | 0.78 (0.70-0.88) |
| Additionally adjusted for parental asthma | 14,284 | 0.78 (0.69-0.88) |
| Complete-case analysis | 8,076 | 0.78 (0.66-0.93) |
| Only among children without parental diabetes at childbirth | 13,227 | 0.78 (0.69-0.88) |
| Full siblings | 13,129 | 0.76 (0.66-0.86) |
| Only among siblings within 5 years of birth year gap | 12,301 | 0.76 (0.66-0.87) |

OR: odds ratio; CI: confidence interval

Models were conditioning on sibling groups, and were adjusted for maternal education, paternal education, maternal age at delivery, maternal body mass index during pregnancy, maternal history of diabetes (when appropriate), paternal history of diabetes (when appropriate), birth order, gestational age and birth weight for gestational age. Models were additionally adjusted for maternal marital status, mode of delivery and parental asthma, when appropriate.
